# Systemic lupus erythematosus complicated by refractory *Mycobacterium avium complex* infection involving bilateral lower extremities: a case report and literature review

**DOI:** 10.3389/fmed.2026.1936197

**Published:** 2026-09-14

**Authors:** Yuanyuan Liu, Yulin Liu, Shengxun Huang, Yanan Zhao, Li Zhu, Tiantian Wang, Hongbin Liang, Mengliu Liu, Wei Geng

**Affiliations:** 1 Department of Orthopaedics, Baoding No.1 Central Hospital, Baoding, Hebei, China; 2 Baoding Key Laboratory of Mechanisms and Integrated Chinese and Western Medicine Interventions for Orthopedic Perioperative Complications, Baoding, Hebei, China

**Keywords:** metagenomic next-generation sequencing, *Mycobacterium avium complex*, nontuberculous mycobacteria, osteomyelitis, systemic lupus erythematosus

## Abstract

**Background:**

Patients with systemic lupus erythematosus (SLE) receiving long-term glucocorticoids and immunosuppressants are at markedly increased risk of opportunistic infections. *Mycobacterium avium complex (MAC)* is a common nontuberculous mycobacterial pathogen, but MAC osteomyelitis is extremely rare in non-HIV SLE patients, posing diagnostic and therapeutic challenges.

**Case presentation:**

We report a 52-year-old female with SLE on long-term prednisone, azathioprine, leflunomide, and hydroxychloroquine, who developed bilateral tibial and femoral osteomyelitis complicated by soft tissue abscesses, drug-induced dermatitis, and severe malnutrition. Conventional microbiological tests remained negative. Metagenomic next-generation sequencing (mNGS) of lower-extremity secretions and pus identified MAC. She underwent five surgical debridements and prolonged multidrug antimycobacterial therapy (azithromycin, rifampicin, ethambutol, amikacin), along with nutritional support and immunosuppressant adjustment. The infection was controlled with no SLE flare, and she recovered favorably.

**Conclusion:**

Disseminated MAC osteomyelitis can occur in SLE patients under sustained immunosuppression even during disease remission. mNGS enables rapid pathogen identification and is crucial for diagnosing complicated infections. Management requires combined surgical debridement, prolonged antimycobacterial therapy, and multidisciplinary care.

## Introduction

Systemic lupus erythematosus (SLE) is a chronic autoimmune disease characterized by immune complex deposition and multi-system involvement. Patients with SLE experience a markedly increased risk of opportunistic infections due to immune dysfunction caused by the disease itself and long-term treatment with glucocorticoids and immunosuppressants (1). The incidence of non-tuberculous mycobacterial infections in immunocompromised populations is increasing year by year, among which *Mycobacterium avium complex* (MAC) is one of the primary pathogens causing pulmonary and disseminated infections (2). However, MAC-induced osteomyelitis is extremely rare, particularly in non-HIV patients, for whom only a handful of cases have been reported (3).

Long-term use of glucocorticoids and immunosuppressants (e.g., azathioprine and leflunomide) can impair the host's defense against intracellular pathogens by inhibiting macrophage activation and T-cell function (5). In patients with SLE, MAC infections primarily manifest as localized skin or soft tissue lesions, while cases progressing to disseminated infections or osteomyelitis are only sporadically reported in individual case reports (4). Due to the slow growth of MAC, low positive rate of conventional culture, and non-specific clinical manifestations, it is extremely prone to misdiagnoses or missed diagnoses in clinical practice, leading to delayed treatment and poor prognosis (3).

This article reports a 52-year-old female patient with long-term SLE who developed bilateral tibial and femoral osteomyelitis successively under maintenance treatment with multiple immunosuppressants, and was diagnosed with MAC infection through metagenomic next-generation sequencing (mNGS). During the course of the disease, the patient developed drug-induced dermatitis and severe malnutrition, and finally achieved a satisfactory outcome after multidisciplinary collaborative treatment. This case report aims to improve the understanding of atypical mycobacterial bone infections in immunocompromised populations and explore the clinical value of the mNGS technique in the identification of difficult-to-diagnose infection pathogens.

## Case presentation

A 52-year-old female patient was admitted to the hospital due to “pain in the proximal left calf for 8 days”. She had a 19-year history of systemic lupus erythematosus. Previously, as her condition relapsed when prednisone was reduced to 12.5 mg/day, she was under long-term maintenance treatment with prednisone at 15 mg/day, hydroxychloroquine sulfate at 0.2 g/day, leflunomide at 10 mg/day, and azathioprine at 100 mg/day. Meanwhile, she had a 2-year history of type 2 diabetes mellitus, and her blood glucose was poorly controlled with oral hypoglycemic drugs. She underwent bilateral hip decompression surgery 17 years ago due to steroid-induced avascular necrosis of the femoral head. Eight days before admission, she developed swelling and pain in the medial proximal left calf without obvious triggers. Imaging examination in another hospital suggested possible osteonecrosis of the proximal left tibia, accompanied by soft tissue inflammatory changes. Upon admission, the patient's condition progressively aggravated, and she developed local suppuration of the left calf, drug-induced dermatitis (flushing of the face and scattered erythema all over the body after infusion of norvancomycin), recurrent fever, and substantial weight loss (from 61.5 kg to 58.5 kg within one month, with BMI decreasing from 23.5 kg/m^2^ to 22.3 kg/m^2^) successively. In Week 3 of the disease course, swelling and fluctuating sensation appeared in the posterior right thigh and popliteal fossa, indicating that the infection had spread to the contralateral lower extremity.

Upon admission, physical examination showed: local swelling (approximately 4 cm in diameter), increased skin temperature, and tenderness on the medial side of the proximal left tibia, with slightly limited flexion and extension of the left knee joint. Laboratory tests indicated: white blood cell count: 9.64 × 10⁹/L; neutrophil count: 7.85 × 10⁹/L; lymphocyte count: 0.92 × 10⁹/L; erythrocyte sedimentation rate: 95 mm/h; C-reactive protein: 109.8 mg/L; procalcitonin: 0.077 ng/mL. Immunological tests suggested: positive antinuclear antibody (titer 1:100-1:320), anti-SS-A antibody (++), anti-Ro-52 antibody (+++), and anti-ribosomal P protein antibody (+++). Complement C3 was at the upper limit of normal, and C4 was at 0.25 g/L. The Department of Rheumatology and Immunology assessed the patient as having “no evidence of lupus flare”. *Mycobacterium avium complex* (MAC) was detected through pathogen metagenomic sequencing of the pus aspirated from the left lower extremity, with Torque teno virus (a commensal virus) detected in the human microbiome simultaneously. At the same time, MAC was also detected by mNGS of pus aspirated from the right popliteal fossa, suggesting possible contralateral involvement along with Torque teno virus and JC polyomavirus (clinically considered commensal or conditionally pathogenic). Pathological results of the left calf tissue (23 April 2026): Fibrous tissue and degenerated necrotic bone tissue were observed, with necrosis, granulation tissue, massive infiltration of acute and chronic inflammatory cells, and local spindle cell proliferation. Immunohistochemical examination of the left calf tissue: Fibrous tissue and degenerated necrotic bone tissue showed necrosis, granulation tissue, massive infiltration of acute and chronic inflammatory cells, and local spindle cell proliferation, considered as proliferative fibrous tissue. Immunohistochemistry results: CK (-), S-100 (-), SMA (-), Desmin (-), SOX10 (-), Ki-67 (5%), SATB2 (-), CD117 (-), CD34 (-), CD68 (+), CD163 (+), CD99 (partially +) (Figures 1, 2). Formal bone culture and drug susceptibility testing for mycobacteria were not performed due to the prolonged turnaround time and the patient's urgent need for treatment. However, the diagnosis was firmly established by two independent mNGS assays. Imaging examinations: Plain CT scan of the left lower extremity (30 March 2026) showed cortical bone destruction and periosteal reaction in the distal left femur and proximal left tibia, with a soft tissue mass adjacent to the proximal tibia, providing detailed osseous structural information (Figure 3). MRI of the left lower extremity (01 April 2026) further characterized the extent of bone marrow edema, soft tissue abscess formation, and the relationship of the lesion to adjacent muscles and joints, indicating bilateral tibial and femoral osteonecrosis with soft tissue infection (Figure 4). PET-CT (30 April 2026) revealed hypermetabolic foci in the muscle spaces of both calves and thighs, offering complementary information on disease activity and systemic distribution of infectious lesions (Figure 5). MRI of the right lower extremity (21 May 2026) confirmed contralateral bone involvement, showing irregular local bone morphology of the femur and tibia, with multiple irregular patchy mixed high and low signals on T1WI and T2WI, along with unclear boundaries (Figure 6). Plain CT scan of the right lower extremity (24 May 2026) further delineated the structural changes on the contralateral side, including multiple patchy slightly low-density shadows in the distal femur and proximal tibia with high-density margins, a small amount of joint effusion, and a low-density shadow in the popliteal fossa, which together confirmed bilateral osteomyelitis and guided the subsequent surgical approach (Figure 7).

**Figure 1:**
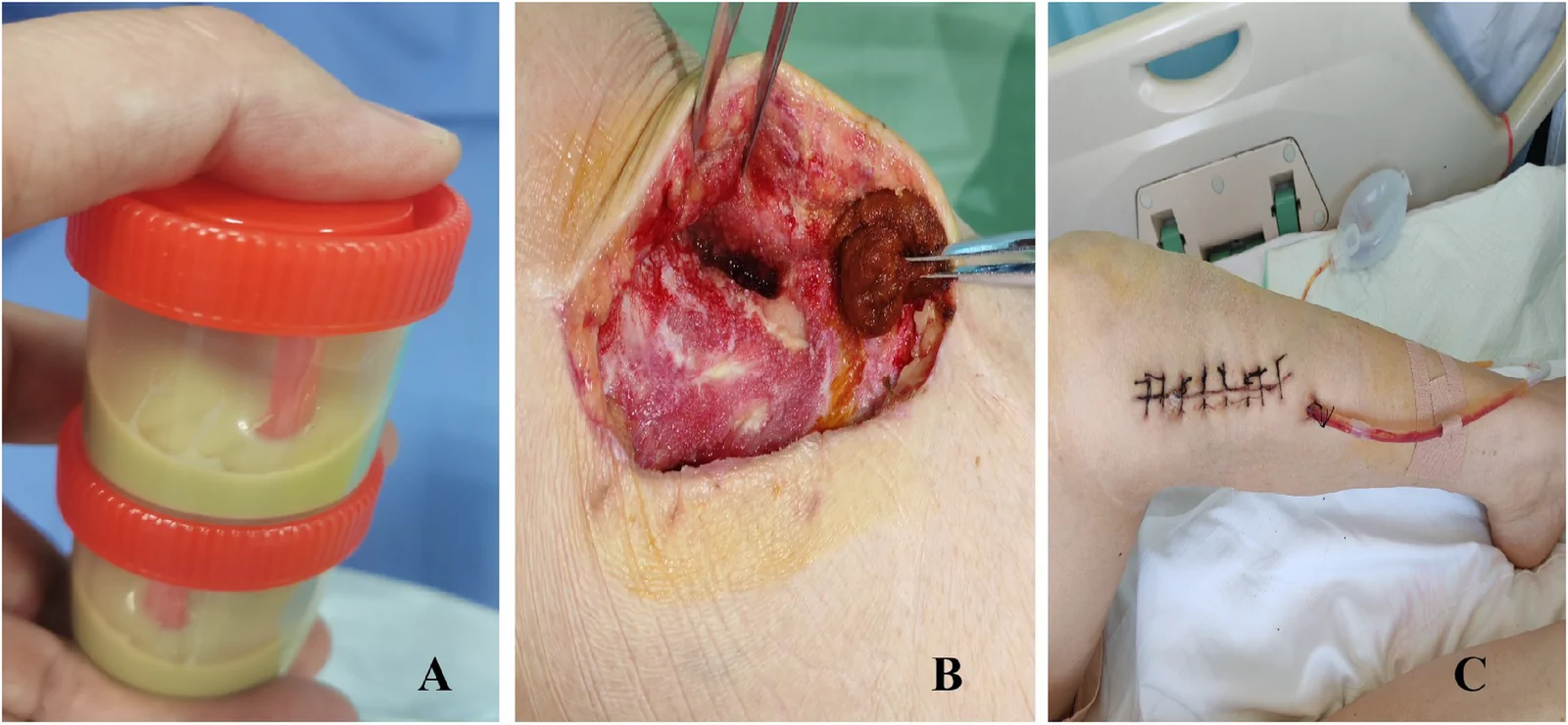
**(A)** A large amount of viscous pus aspirated from the left lower extremity under ultrasound guidance; **(B)** incision and drainage wound of the left lower extremity; **(C)** postoperative state of left lower extremity incision and drainage.

**Figure 2:**
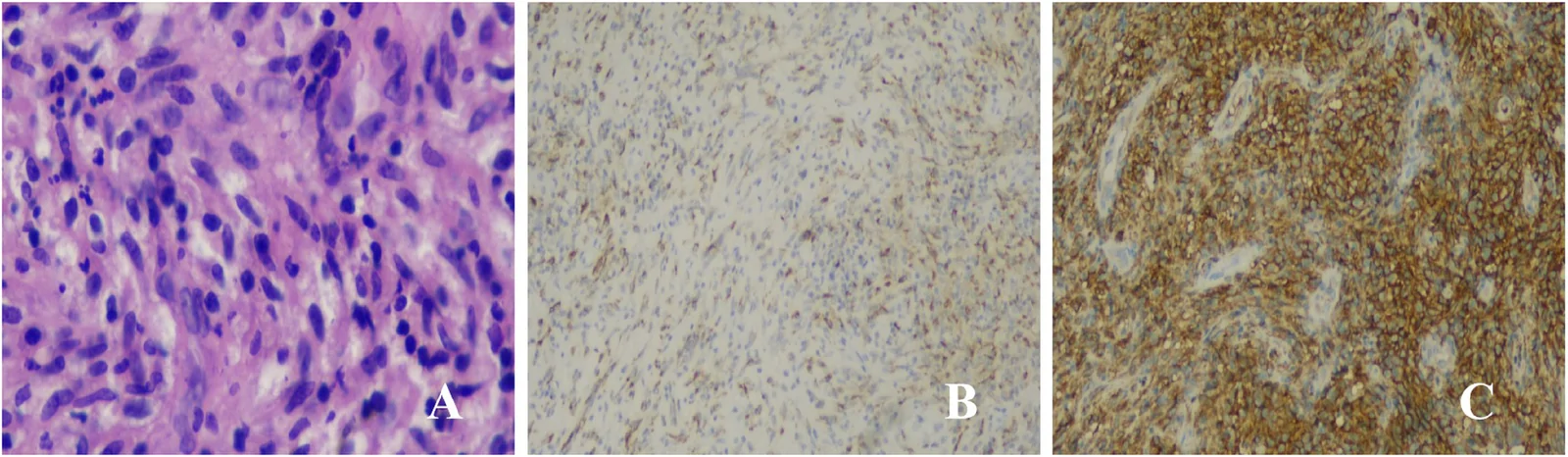
**(A)** pathological results of left calf tissue: fibrous tissue and degenerated necrotic bone tissue, with necrosis, granulation tissue, massive infiltration of acute and chronic inflammatory cells, and local spindle cell proliferation (HE staining   ×  100). **(B,C)** Immunohistochemical examination of left calf tissue: Fibrous tissue and degenerated necrotic bone tissue, with necrosis, granulation tissue, massive infiltration of acute and chronic inflammatory cells, and local spindle cell proliferation, considered as proliferative fibrous tissue. Immunohistochemistry results: CK (-), S-100 (-), SMA (-), Desmin (-), SOX10 (-), Ki-67 (5%), SATB2 (-), CD117 (-), CD34 (-), CD68 (+), CD163 (+), CD99 (partially+) (×100).

**Figure 3:**
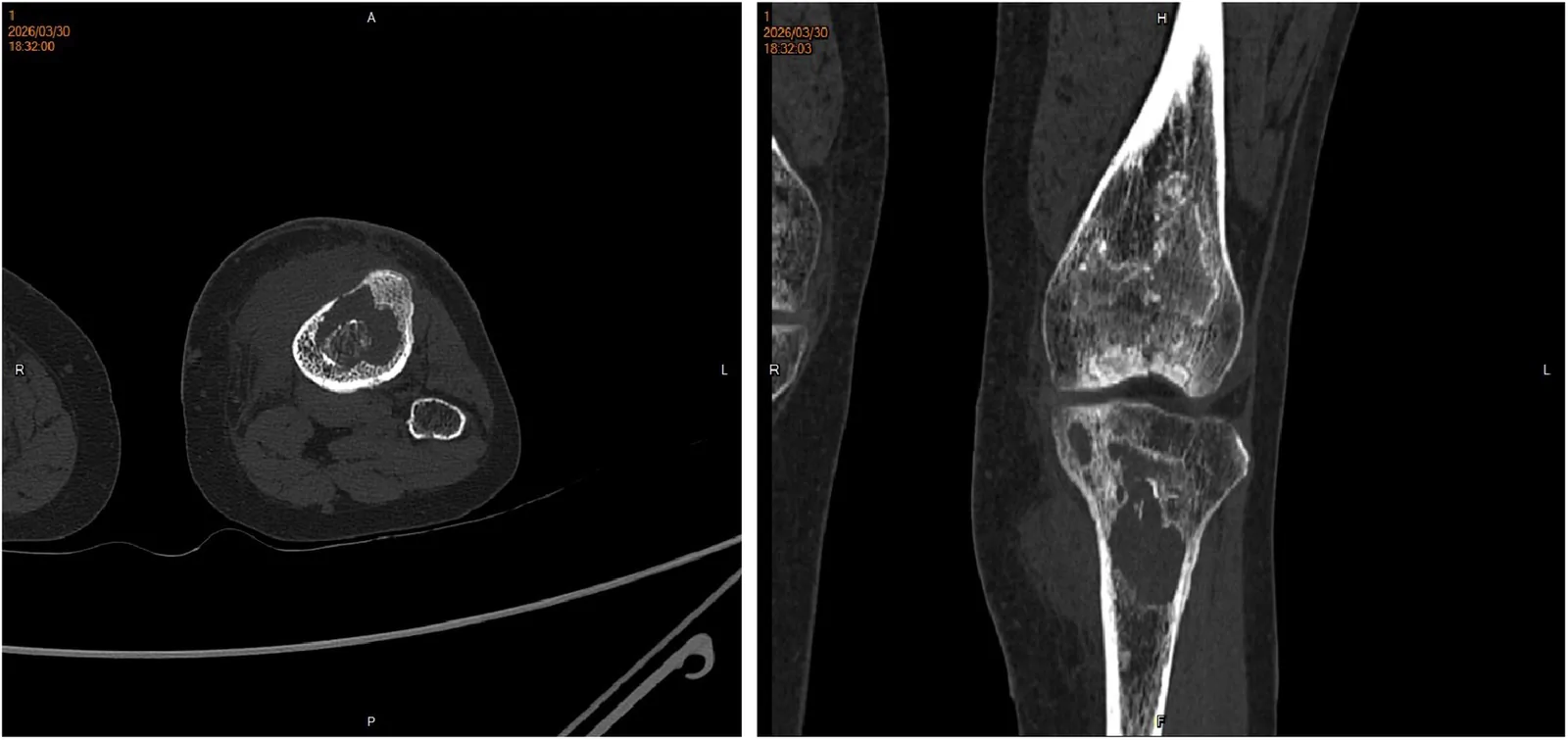
Plain CT scan of the left lower extremity: abnormal bone changes in the distal left femur and proximal left tibia, and a soft tissue mass adjacent to the proximal tibia.

**Figure 4:**
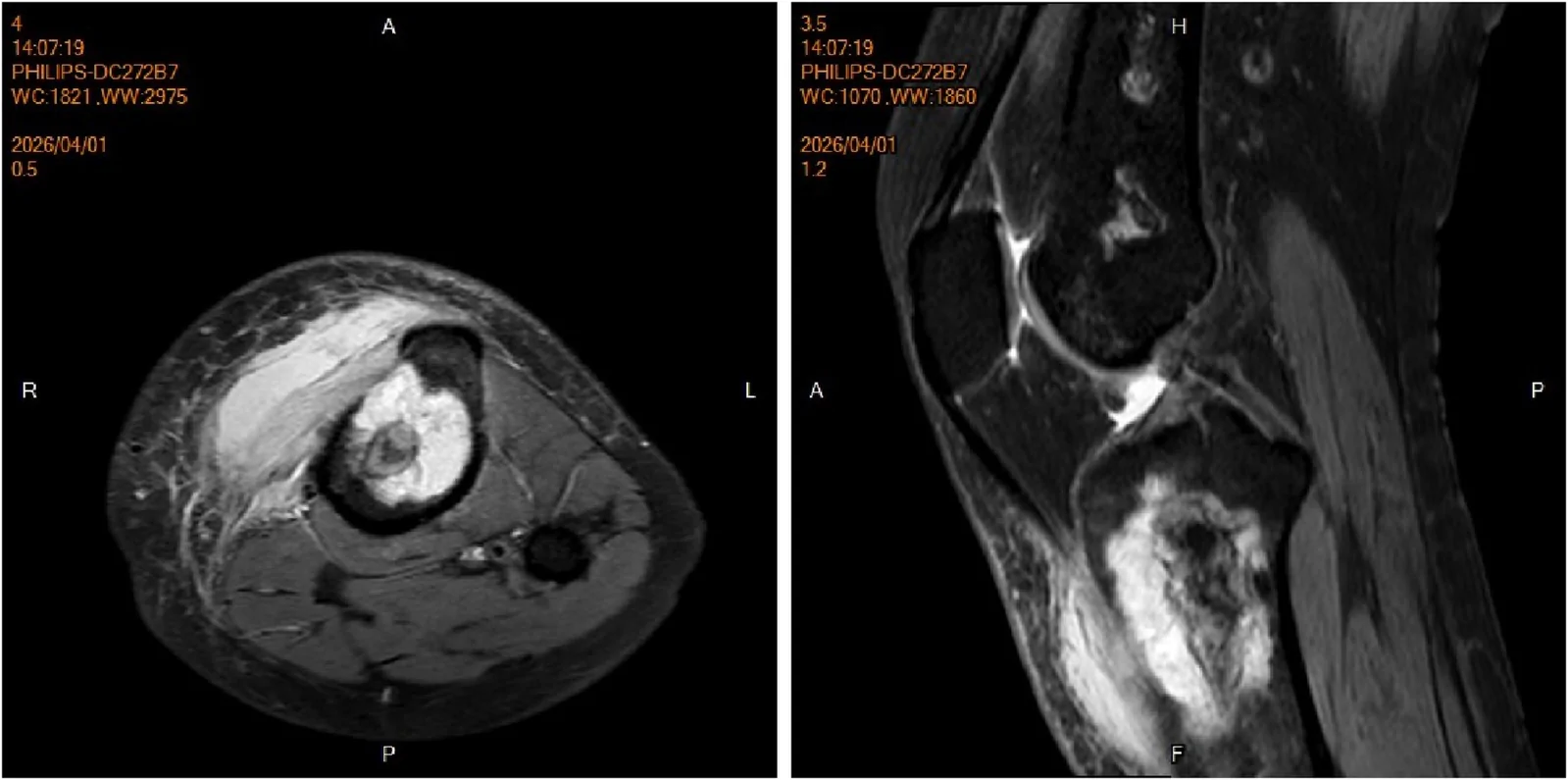
MRI of the left lower extremity: irregular local bone morphology of the distal left femur and proximal left tibia, and multiple irregular patchy mixed high and low signals on T1WI and T2WI, with unclear boundaries. A mass-like lesion with low signal on T1WI and high signal on T2WI visible on the medial side of the proximal tibia, with unclear boundaries, measuring approximately 4.6 × 4.7 × 2.9 cm. On enhanced scan, the periphery shows obvious enhancement, with no obvious enhancement in the central part, and the damaged adjacent tibial cortex is visible. Flocculent high signals on PDWI are noted in the subcutaneous soft tissue. Bone infarction in the distal left femur and proximal left tibia complicated by soft tissue infection in the proximal tibia is considered.

**Figure 5:**
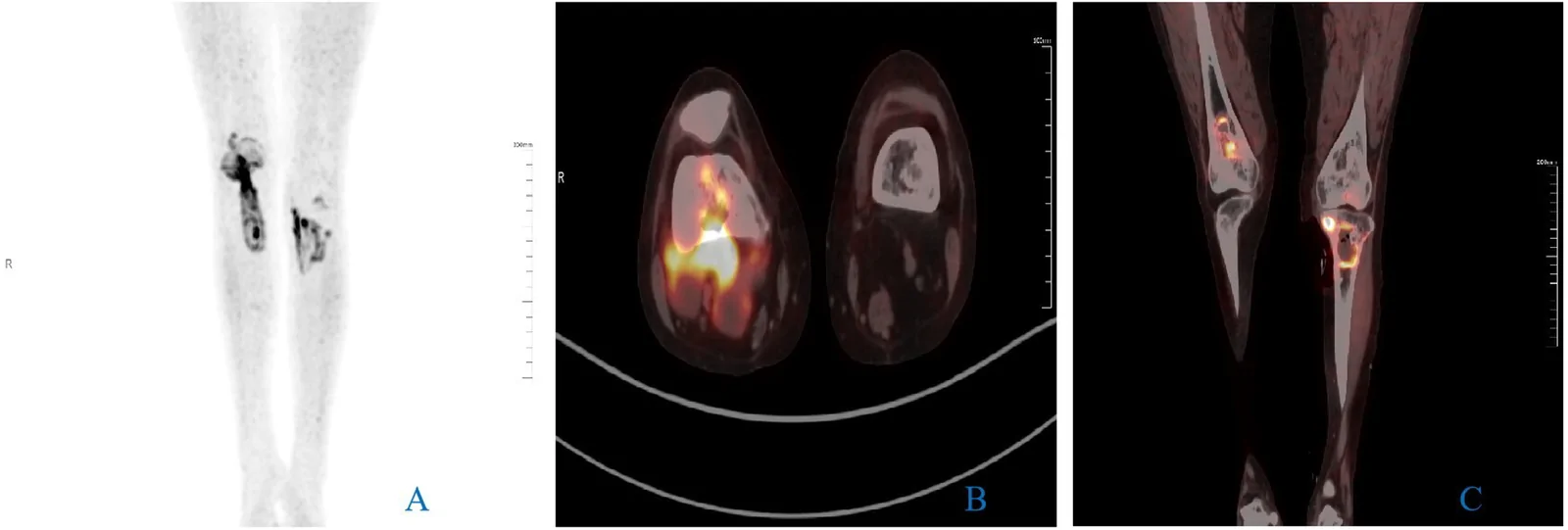
PET-CT findings: PET-CT findings of the lower extremities. **(A)** Postoperative changes in the left calf and proximal left tibia, showing heterogeneous increased fluorodeoxyglucose (FDG) uptake in the surgical area. **(B)** Hypermetabolic foci in the proximal left tibia and distal bilateral femurs, consistent with active infectious lesions. Multiple low-density foci with mildly increased FDG uptake in the distal bilateral femurs and proximal tibias are also observed. **(C)** A thick-walled low-density lesion with ring-shaped increased FDG uptake in the intermuscular space between the distal right thigh and proximal right calf, suggestive of an infectious process. Bilateral femoral head necrosis is also noted.

**Figure 6:**
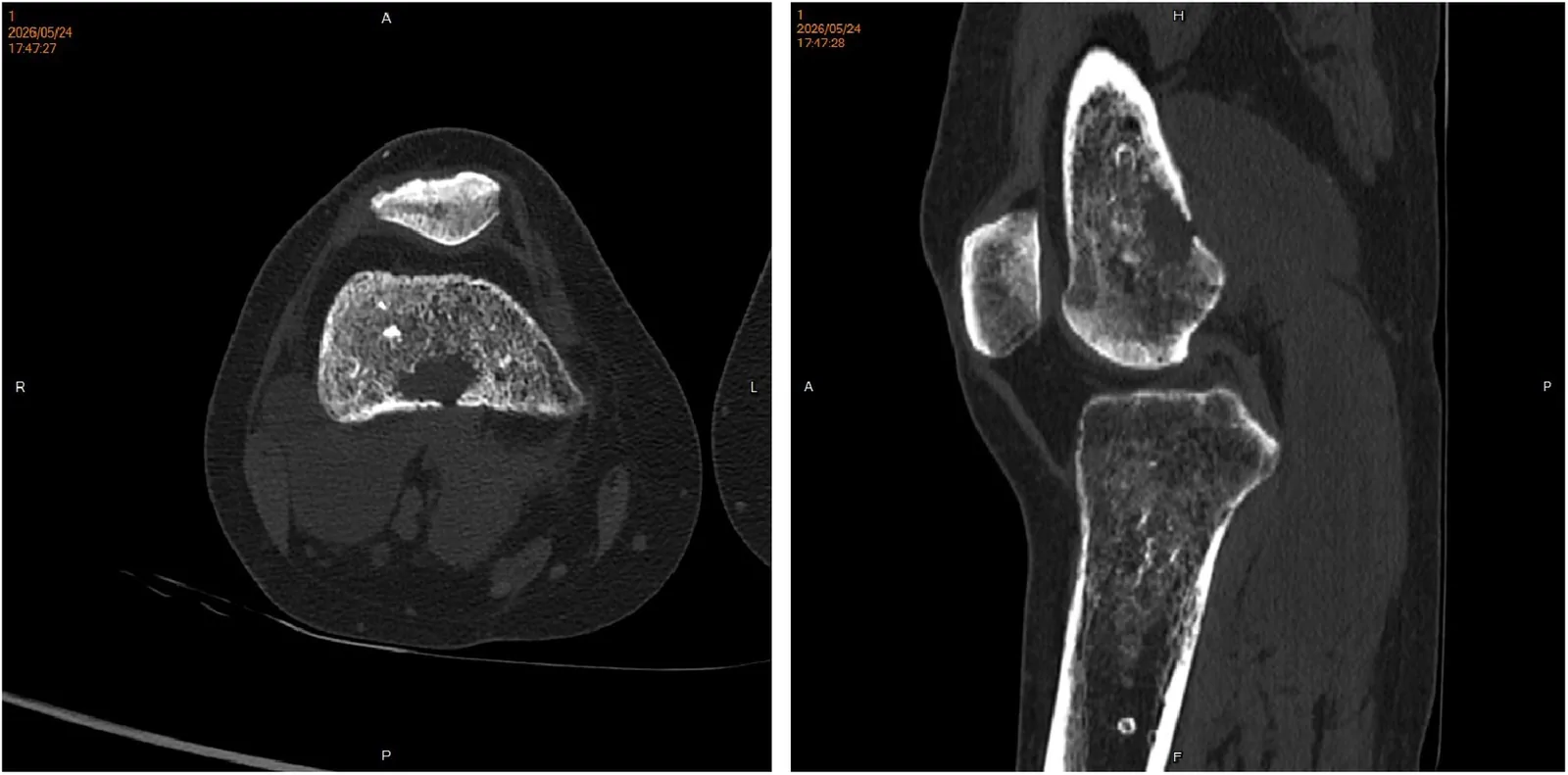
CT findings of the right lower extremity: well-aligned right knee joint; and multiple patchy slightly low-density shadows with high-density edges visible in the distal femur and proximal tibia. A small amount of fluid density shadow is noted in the joint capsule. A slightly low-density shadow is visible in the popliteal fossa.

**Figure 7:**
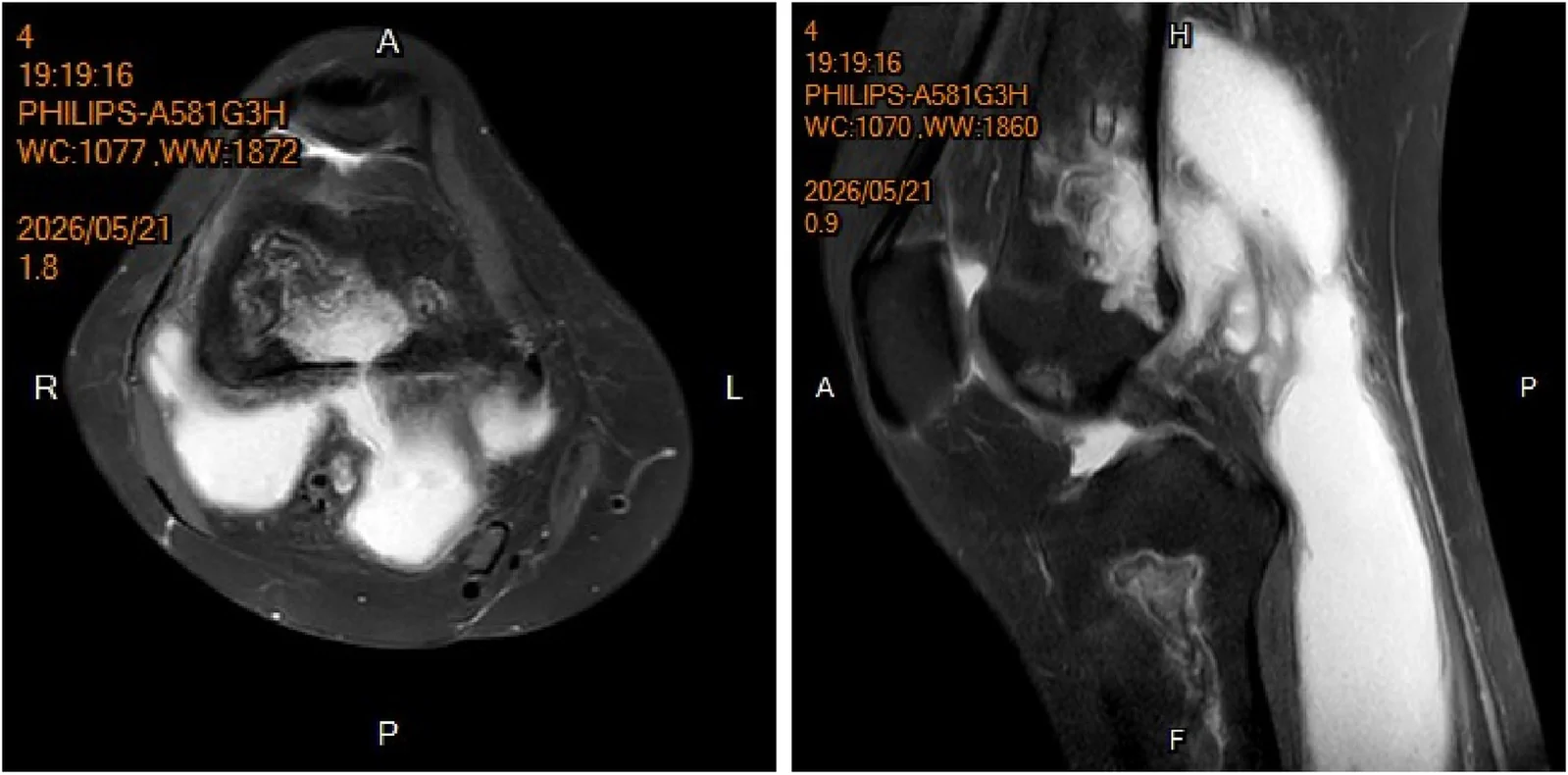
MRI findings of the right lower extremity: well-aligned right knee joint; irregular local bone morphology of the femur and tibia; multiple irregular patchy mixed high and low signals on T1WI and T2WI visible; and unclear boundaries. Bone infarction in the right femur and tibia is highly suspected.

In terms of treatment, the patient underwent 5 surgeries successively, including incision and drainage of the left calf abscess, muscle debridement, bone trough fenestration, bone cement filling, and debridement of the right femoral osteomyelitis lesion, with thickened periosteum, bone abscess cavities, and a large amount of viscous pus was observed intraoperatively (Figure 1). As for anti-infection therapy, initial empirical treatment was ineffective. After the mNGS results were available, an anti-mycobacterial regimen (azithromycin + rifampicin + ethambutol + amikacin) was initiated, with postoperative fever gradually controlled. Meanwhile, comorbidities were managed as follows: the Endocrinology Department adjusted the hypoglycemic regimen; the Nutrition Department assessed the patient as suffering “severe malnutrition” and provided enteral nutrition support (target energy: 1700 kcal/d); the Department of Rheumatology and Immunology reduced the dose of prednisone and discontinued azathioprine. After nearly 2 months of comprehensive treatment, the patient's body temperature returned to normal, inflammatory markers decreased substantially (C-reactive protein dropped to below 30 mg/L), and the wounds on both lower extremities healed well. At discharge, her weight was 60.2 kg, with a BMI of 22.9 kg/m^2^. The patient was transferred to a specialized hospital for continued anti-mycobacterial treatment, and telephone follow-ups through early June 2026 showed that the patient had no fever or recurrent lupus and could walk with crutches. A chronological summary of the patient's clinical course, diagnostic procedures, and therapeutic interventions is presented in Figure 8.

**Figure 8:**
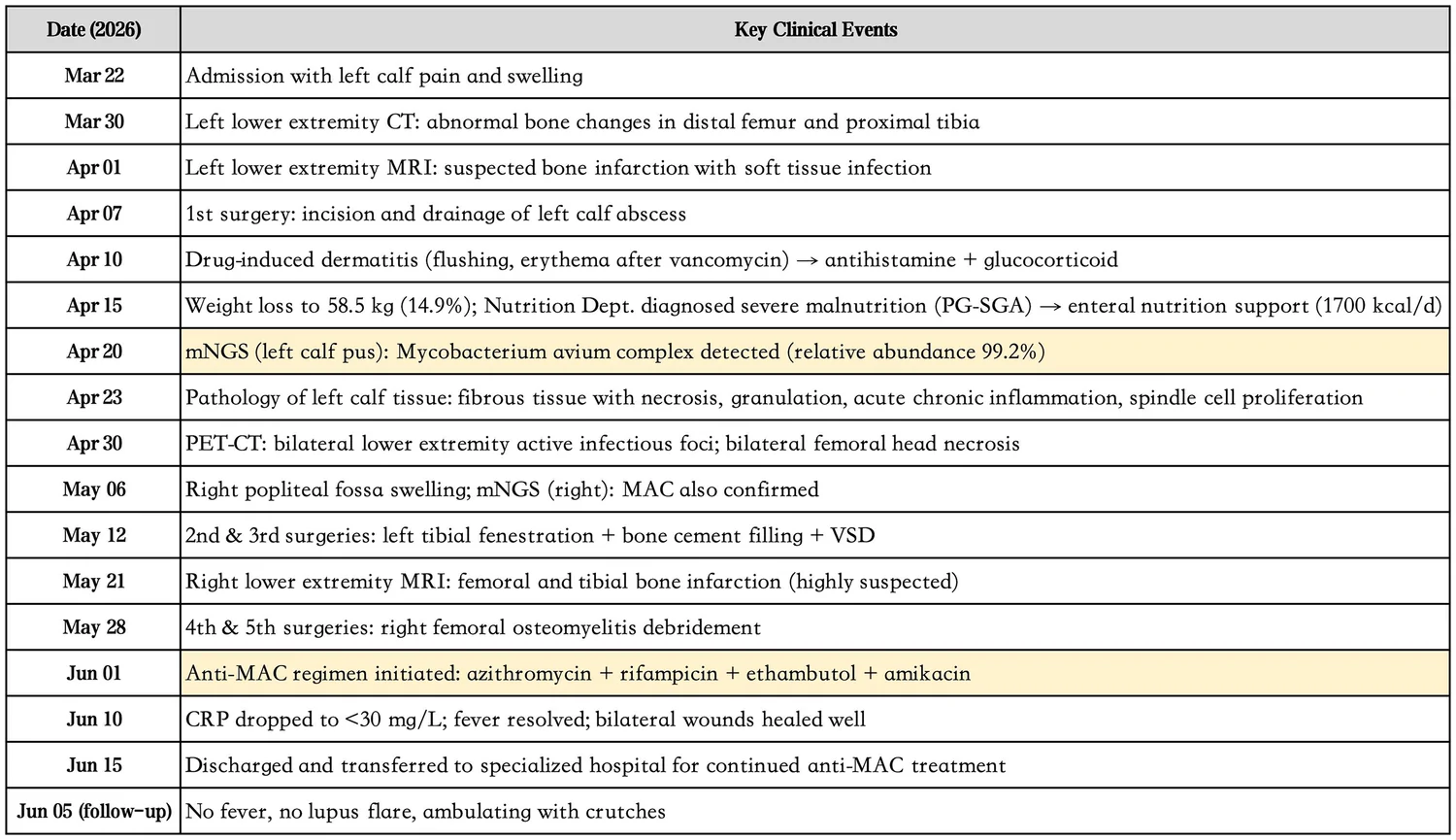
Chronological timeline of the patient's disease course and major interventions. (Baoding No.1 Central Hospital, 2026). Chronological timeline of the patient's disease progression and management. Key events include admission, imaging examinations, five surgical debridements, mNGS-based pathogen identification, anti-MAC regimen initiation, nutritional support, immunosuppressant adjustment, and discharge. MAC, *Mycobacterium avium complex*; mNGS, metagenomic next-generation sequencing; CRP, C-reactive protein; PG-SGA, Patient-Generated Subjective Global Assessment; VSD, vacuum sealing drainage.

## Discussion

This case report describes a 52-year-old female patient with long-term SLE who developed bilateral tibiofemoral osteomyelitis and disseminated soft tissue infection caused by Mycobacterium avium complex (MAC) under maintenance immunosuppressive therapy. Three aspects of this case merit particular attention: the rare occurrence of MAC osteomyelitis in non-HIV SLE patients, the rapid identification of the pathogen by mNGS, and the successful multidisciplinary management of this complex condition.

SLE patients are under a state of long-term immune disorder, and the combined use of glucocorticoids and immunosuppressants makes them susceptible to opportunistic infections (5, 7). The patient in this case report had long-term use of prednisone at 15 mg/day, azathioprine at 100 mg/day, and leflunomide at 10 mg/day, with a relatively high intensity of immunosuppression. Previous studies have shown that long-term use of glucocorticoids (equivalent to prednisone > 7.5 mg/day for over 180 days) can lead to markedly increased risks of severe infection and osteomyelitis in SLE patients (6). A systematic review by Rella V et al. (1) indicated that the risk of osteomyelitis in SLE patients is about 8.5 times that of the general population. This increased susceptibility is generally attributed to the combined effects of disease-related immune dysregulation and prolonged immunosuppressive therapy, although the precise mechanisms remain incompletely understood (1, 5). In this regard, it is worth noting that while this patient was positive for antinuclear antibody and anti-SS-A antibody, complement C3 and C4 were basically normal, and the Department of Rheumatology and Immunology repeatedly assessed her as having “no lupus activity”. This suggests that the infection was not caused by active lupus but more likely related to “impaired host immune defense” associated with long-term immunosuppressive treatment. This phenomenon is similar to the case reported by Gray ME et al. (3), in which an SLE patient with long-term use of prednisone at 20 mg/day also developed MAC spinal osteomyelitis during the stable phase of lupus. Therefore, clinicians should recognize that even in the remission phase of SLE, long-term immunosuppressive treatment itself is an essential independent risk factor for non-tuberculous mycobacterial (NTM) infection.

The clinical manifestations of MAC infection in SLE patients are diverse and non-specific. Yao QH et al. (2), through a systematic review of 21 patients with SLE complicated by primary cutaneous MAC infection, found that the skin manifestations of such patients could manifest as nodules, ulcers, erythema, cold abscesses, and other forms, which were prone to being confused with sporotrichosis, actinomycosis, etc. In this case report, the patient initially presented with local swelling and pain in the left calf, and the initial imaging findings were suspected to be bone tumor. This diagnostic dilemma was also reported in previous literature (7). Given this, the diagnostic challenges of MAC osteomyelitis mainly result from three aspects: 1. MAC grows slowly, and conventional Lowenstein-Jensen medium takes 2–6 weeks to report positive results, which is much longer than that of common pyogenic infections; 2. Acid-fast staining cannot distinguish Mycobacterium tuberculosis from non-tuberculous mycobacteria, and MAC is naturally resistant to most anti-tuberculosis drugs (3); 3. When SLE patients have symptoms such as fever and bone pain, it is necessary to differentiate them from lupus activity, drug fever, other infections, aseptic osteonecrosis, and other conditions. In this case report, the blood culture and routine secretion culture of the patient were negative after admission, and the pathogen was not identified until the cultures were sent for testing via mNGS after surgery, which fully showcases the limitations of conventional microbiological tests in the diagnosis of NTM infection.

The mNGS technique played a decisive role in the diagnosis of the patient in this case report. For this patient, after mNGS was performed twice on her left calf tissue and the posterior part of her right thigh and popliteal fossa, MAC was detected with high confidence in both tests, and the relative abundance of Mycobacterium in her left calf tissue was up to 99.2%, strongly supporting MAC as the pathogenic pathogen. Compared with conventional culture methods, mNGS offers the advantages of being unbiased, rapid, and highly sensitive, which is especially suitable for the diagnosis of difficult infections in immunocompromised patients (2, 8). In the case reported by Yao QH et al. (2), mNGS also successfully confirmed MAC infection, providing a basis for subsequent targeted therapy. Additionally, viral sequences such as Torque teno virus, JC polyomavirus, EBV, and CMV were also detected through the mNGS performed for the patient in this case report, which were clinically judged to be commensal or conditionally pathogenic. However, this phenomenon is not uncommon in immunosuppressed patients, suggesting that mNGS results need to be comprehensively interpreted in combination with clinical conditions, so as to avoid overtreatment. We acknowledge that Ziehl-Neelsen (ZN) staining was not performed on the bone tissue in this case. This decision was made because the patient's clinical presentation was initially non-specific, and conventional microbiological tests including acid-fast staining were not prioritized at the time of specimen collection. However, the diagnosis of MAC infection was unequivocally established by two independent mNGS assays performed on pus samples from both the left calf and the right popliteal fossa, with high reads and relative abundance (99.2% in the left calf specimen). Considering the high sensitivity and specificity of mNGS for pathogen detection in complicated infections, we believe that the absence of ZN staining does not compromise the diagnostic validity of this case. We also acknowledge that a formal bone biopsy was not performed. Nevertheless, the diagnosis of osteomyelitis was supported by comprehensive imaging findings (CT, MRI, PET-CT), intraoperative observation of bone destruction and purulent material, and mNGS identification of MAC from deep pus aspirates. Although bone biopsy remains the preferred method for diagnosing osteomyelitis, the consistent clinical, imaging, and microbiological evidence in this case strongly supported the diagnosis.

As for the treatment of MAC infection, the international guidelines currently recommend a dual therapy or triple therapy based on macrolides combined with ethambutol, with clarithromycin or azithromycin as the core drugs (9). For bone and joint infections, due to factors such as poor blood supply at the lesion site, biofilm formation, and bacterial resistance, simple drug therapy is often difficult to eradicate the pathogen, and surgical debridement is required in combination (3, 10). The patient in this case report underwent 5 surgical debridements, including cortical fenestration, bone cement filling, and VSD negative pressure suction, and was treated with a combination regimen of azithromycin + rifampicin + ethambutol + amikacin postoperatively to finally achieve clinical control. This treatment strategy is consistent with literature reports: the patient with MAC spinal osteomyelitis reported by Gray ME et al. (3) achieved complete symptom relief after 12 months of treatment with ethambutol + rifampicin; the patient with SLE complicated by MAC osteomyelitis reported by Yazisiz V et al. (11) was also treated with clarithromycin + ethambutol + ciprofloxacin, and achieved a good outcome in combination with surgical debridement. It is worth noting that rifabutin was initially intended to be used in this case, but was changed to rifampicin due to accessibility. Despite rifabutin has superior *in vitro* activity against MAC compared with rifampicin, the rifampicin-based combination regimen is still widely used in clinical practice (9). Moreover, the course of anti-MAC treatment usually requires over 12 months, and the patient in this case report is still receiving treatment, with long-term follow-up needed to evaluate the long-term efficacy.

The treatment course was further complicated by drug-induced dermatitis and severe malnutrition. The patient developed extensive rash after norvancomycin infusion, which resolved after drug discontinuation and glucocorticoid administration. This underscores the need for cautious drug selection and close monitoring of adverse reactions in SLE patients, who have a higher incidence of drug allergies. Nutritionally, the patient's weight dropped by 4.9% within one month and was assessed as severe malnutrition (PG-SGA), which not only impairs wound healing but also compromises immune function, creating a vicious cycle of “infection–catabolism–immunosuppression" (12). Prompt nutritional intervention (enteral nutrition at 1,700 kcal/d) was crucial for surgical tolerance and infection control.

Compared with previously reported cases of SLE complicated by MAC infection, this case exhibits the following characteristics: 1. The scope of lesion is more extensive, involving the bones and soft tissues of both lower extremities, while most reported cases are localized skin infections or single-focal osteomyelitis (2, 11); 2. The diagnostic process was more complicated, reflecting the complexity of diagnosing difficult infections; 3. Complications such as drug-induced dermatitis and severe malnutrition occurred during treatment, increasing the difficulty of treatment, and the successful practice of multidisciplinary collaboration was one of the features of this case. However, this case also has some limitations: Drug susceptibility test results were not available due to the prolonged culture period of MAC, and the selection of anti-infection regimen was mainly based on literature recommendations and clinical experience; since the current follow-up time is short, further observation is required for the long-term recurrence and the degree of joint function recovery.

## Conclusion

In conclusion, SLE patients experience markedly increased risks of non-tuberculous mycobacterial infections such as MAC after long-term treatment with glucocorticoids and immunosuppressants. The clinical manifestations of MAC osteomyelitis are atypical, and conventional microbiological tests are time-consuming and exhibit low positive rates. By contrast, the mNGS technique can substantially shorten the diagnostic cycle and should be used as a preferred diagnostic method for difficult infections in immunosuppressed populations. In this regard, a comprehensive strategy of “surgical debridement + long-term combined anti-mycobacterial treatment” is required for treatment, while attention should be paid to nutritional support and sophisticated adjustment of immunosuppressants. In view of all the above, this case provides a clinical reference for the diagnosis and treatment of SLE complicated by NTM infection, and more cases need to be included in the future, so as to establish standardized diagnosis and treatment procedures.

## Data Availability

The raw data supporting the conclusions of this article will be made available by the authors, without undue reservation.
