# Correction: Systemic lupus erythematosus complicated by refractory *Mycobacterium avium complex* infection involving bilateral lower extremities: a case report and literature review

**DOI:** 10.3389/fmed.2026.1999626

**Published:** 2026-09-29

**Authors:** Yuanyuan Liu, Yulin Liu, Shengxun Huang, Yanan Zhao, Li Zhu, Tiantian Wang, Hongbin Liang, Mengliu Liu, Wei Geng

**Affiliations:** 1 Department of Orthopaedics, Baoding No.1 Central Hospital, Baoding, Hebei, China; 2 Baoding Key Laboratory of Mechanisms and Integrated Chinese and Western Medicine Interventions for Orthopedic Perioperative Complications, Baoding, Hebei, China

**Keywords:** metagenomic next-generation sequencing, *Mycobacterium avium complex*, nontuberculous mycobacteria, osteomyelitis, systemic lupus erythematosus

Affiliation ¨Baoding Key Laboratory of Mechanisms and Integrated Chinese and Western Medicine Interventions for Orthopedic Perioperative Complications, Baoding, Hebei, China was erroneously omitted for author Yuanyuan Liu, Yulin Liu, Shengxun Huang, Yanan Zhao, Li Zhu, Tiantian Wang, Hongbin Liang, Mengliu Liu and Wei Geng.

The funder ¨The Scientific Research Plan Project of Hebei Provincial Administration of Traditional Chinese Medicine, (No. 2024450) ¨ was erroneously omitted.

The corrected Funding statement reads:

¨The author(s) declared that financial support was received for this work and/or its publication. This work was supported by The Scientific Research Plan Project of Hebei Provincial Administration of Traditional Chinese Medicine, (No. 2024450). ¨

The original version of this article has been updated.

